# Intrinsic Exercise Capacity Affects Glycine and Angiotensin-Converting Enzyme 2 (ACE2) Levels in Sedentary and Exercise Trained Rats

**DOI:** 10.3390/metabo12060548

**Published:** 2022-06-15

**Authors:** Nora Klöting, Michael Schwarzer, Estelle Heyne, Uta Ceglarek, Anne Hoffmann, Knut Krohn, Torsten Doenst, Matthias Blüher

**Affiliations:** 1 Helmholtz Institute for Metabolic, Obesity and Vascular Research (HI-MAG) of the Helmholtz Zentrum München, University of Leipzig, Ph. Rosenthal Street 27, 04103 Leipzig, Germany; anne.hoffmann@helmholtz-muenchen.de (A.H.); matthias.blueher@medizin.uni-leipzig.de (M.B.); 2 Department of Cardiothoracic Surgery, University Hospital Jena, Am Klinikum 1, 07747 Jena, Germany; michael.schwarzer@med.uni-jena.de (M.S.); estelle.heyne@med.uni-jena.de (E.H.); torsten.doenst@med.uni-jena.de (T.D.); 3 Institute of Laboratory Medicine, Clinical Chemistry and Molecular Diagnostics, Leipzig University Medical Center, 04103 Leipzig, Germany; uta.ceglarek@medizin.uni-leipzig.de; 4 CoreUnit DNA Technologies, Medical Faculty, University of Leipzig, Liebigstr. 21, 04103 Leipzig, Germany; knut.krohn@medizin.uni-leipzig.de; 5 Medical Department III—Endocrinology, Nephrology, Rheumatology, University of Leipzig Medical Center, Liebigstr. 20, 04103 Leipzig, Germany

**Keywords:** ACE2, HCR, LCR, exercise capacity, SARS-CoV-2, metabolome

## Abstract

Angiotensin-converting enzyme 2 (ACE2) has been identified as the cellular entry receptor for the novel severe acute respiratory syndrome coronavirus 2 (SARS-CoV-2). High ACE2 tissue expression and low glycine levels were suggested to increase susceptibility for SARS-CoV-2 infection and increasing circulating ACE2 has been proposed as one possible strategy to combat COVID-19. In humans, aerobic physical exercise induces an increase in plasma ACE2 in some individuals. However, it is not clear whether glycine and ACE2 levels depend on intrinsic exercise capacity or on exercise training. We used rats selectively bred for high intrinsic exercise capacity (HCR) or low exercise capacity (LCR) and tested the influence of this genetic predetermination and/or aerobic exercise on metabolites, ACE2 tissue expression and circulating ACE 2. ACE2 expression was measured in different tissues in the sedentary animals and again after 4 weeks of high-intensity aerobic exercise in both LCRs and HCRs. Sedentary HCRs exhibited significantly higher circulating ACE2 concentrations compared to LCRs, but a lower expression of ACE2 in all investigated tissues except for adipose tissue. Body weight was negatively correlated with serum ACE2 and positively correlated with ACE2 expression in the heart. Aerobic exercise caused a significant decrease in ACE2 expression in the lung, heart, muscle, and kidney both in LCRs and HCRs. Our results suggest that ACE2 expression, circulating ACE2 and glycine serum concentration are related to aerobic intrinsic exercise capacity and can be influenced with exercise. These results may support the hypothesis that physically fit individuals have a lower susceptibility for COVID-19 infection.

## 1. Introduction

The current COVID-19 pandemic has mainly affected elderly people with predisposing conditions such as hypertension, diabetes, coronary heart disease, chronic obstructive pulmonary disease, and kidney dysfunction, for which worse clinical outcomes of SARS-coronavirus-2 (SARS-CoV-2) infections have been reported [1,2]. Furthermore, obesity and diabetes have been described as risk factors for SARS-CoV-2 infection as well [1,3]. All these conditions are frequently associated with lower physical fitness and activity.

Angiotensin-converting enzyme 2 (ACE2) was identified as the entry receptor for SARS-CoV-2 [4,5]. Based on the concept that increasing circulating ACE2 may protect against COVID-19 by occupying the cellular entry receptors on the virus, recombinant human ACE2 (rhACE2/APN01/GSK2586881) originally developed to fight other acute respiratory distress syndromes will be tested in a clinical trial to treat COVID-19 [6]. Medications that act on the renin–angiotensin–aldosterone system including ACE-inhibitors and angiotensin-II-receptor subtype 1 blockers (ARBs) were suggested to interfere with the regulation of ACE2, raising concerns about their use during the pandemic [7]. Moreover, hypertension has emerged as an important COVID-19 comorbid condition [8]. Recent analyses of independent COVID-19 cohorts did not reveal an association between the infection rate or the severity of the disease with the application of ACE-inhibitor, ARBs, or other antihypertensive medications [7,9].

Increased ACE2 plasma concentrations along with an increased urinary excretion of ACE2 after physical exercise were reported in humans [10]. In addition, ACE2 deficiency affects physical performance and impairs cardiac and skeletal muscle adaptations to exercise [11]. Interestingly, ACE2 null mice have significantly decreased glycine levels [12], suggesting that the activation of ACE2 would increase plasma glycine levels. Recently, it was proposed that glycine itself should be used in the treatment of COVID-19 patients due to its anti-inflammatory, anti-oxidative, neurological functions, and role in metabolic regulation [13,14].

Thus, there is a connection between exercise, ACE2, metabolites, and glycine. However, exercise capacity consists of two components: intrinsic (genetically determined) exercise capacity determines the major part of exercise capacity while extrinsic (environmentally determined) exercise capacity plays only a minor role. The influence of both components of exercise capacity on ACE2 expression and glycine is not clear. In humans, the separation of both components of exercise capacity and their effect on ACE2 seems not easily possible due to the plethora of environmental influences. The previously described rat model representing differences in exercise capacity allows for such discrimination [15]. The rats were selectively bred for either high or low intrinsic aerobic exercise capacity (high-capacity runners, HCRs; low-capacity runners, LCRs) and differ in their intrinsic exercise capacity by approximately 800% [16]. Additionally, LCRs develop signs of metabolic syndrome including obesity and may therefore reflect the previously reported COVID-19 higher risk phenotype [2].

We therefore tested the hypotheses that metabolites such as glycine and ACE*2* tissue expression and serum concentrations are related to intrinsic exercise capacity and that exercise training alters ACE*2* tissue expression differently depending on intrinsic exercise capacity.

## 2. Results

### 2.1. HCR Rats Are Leaner and Have Less ACE2 mRNA Tissue Expression

**Figure 1:**
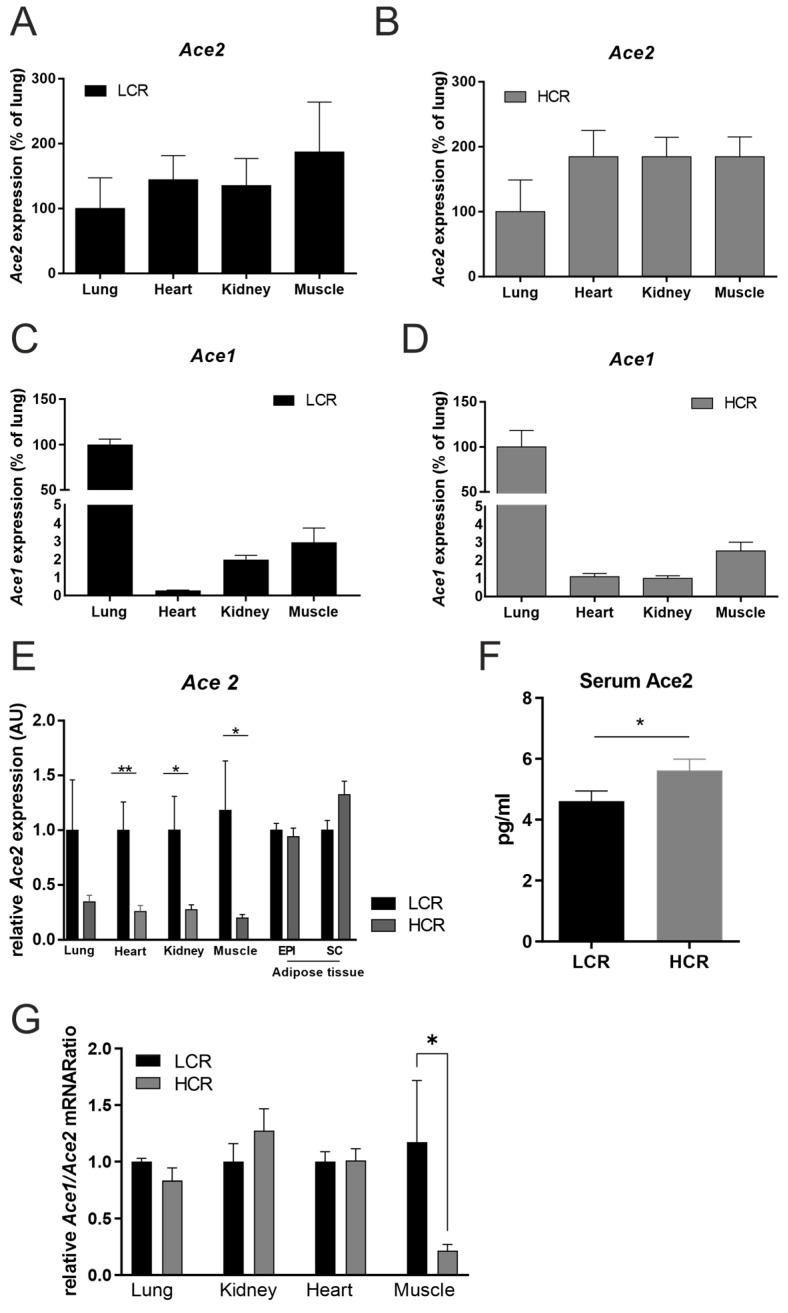
ACE2 and ACE1 mRNA expression in different tissues and ACE2 serum concentration in low- and high-capacity rats. At the age of 19 weeks, 12 rats—either low-capacity runners (LCRs, *n* = 6) or high-capacity runners (HCRs, *n* = 6) were investigated. (**A**) Body weight in LCRs versus HCRs; ACE2 expression in lung, heart, kidney, skeletal muscle (Musculus quadriceps femoris) and adipose tissue epigonadal (EPI) as well as subcutaneous (SC) of (**B**) HCR and (**C**) LCR rats. (**D**) ACE1 mRNA expression in lung, heart, kidney, and skeletal muscle of HCRs. (**E**) Comparison of relative ACE2 mRNA expression between LCRs and HCRs in different tissues. (**F**) ACE2 serum concentrations in LCRs and HCRs. (**G**) The ACE1/ACE2 mRNA ratio in different tissues of LCR and HCR rats. Results are expressed as the means ± SE. The different degrees of significance are indicated as follows: *, *p* < 0.05; **; *p* < 0.01.

Sedentary low-capacity runners (LCRs) were heavier than HCRs (227 g ± 17 g vs. 195 g ± 10 g; *p* = 0.015) and their muscles showed the highest expression of ACE*2*, followed by the heart, kidney and lungs (Figure 1A,B). The tissue distribution of ACE*2* mRNA expression was comparable between the LCR and HCR rats (Figure 1A,B). In contrast, ACE1 expression was highest in the lung, followed by muscle, kidney, and the heart in HCR (Figure 1D) and LCR rats (Figure 1C). Interestingly, ACE2 expression was higher in the LCRs compared to the HCRs in the lung, muscle, kidney, and heart. The most pronounced differences were found in the heart (Figure 1E). Adipose tissue ACE2 expression was not affected by exercise capacity (Figure 1E). In contrast to higher tissue ACE2 mRNA expression, circulating serum ACE2 concentrations were significantly lower in LCR compared to HCR rats (Figure 1F). A significantly lower ACE1/ACE2 mRNA ratio was detected in the muscle of HCR rats (Figure 1G).

### 2.2. Metabolome Analysis

**Figure 2:**
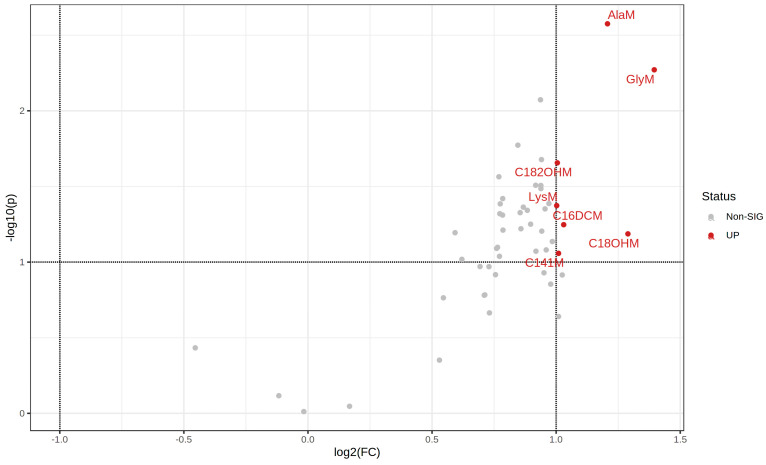
Upregulated metabolites in HCRs compared to LCRs. The metabolites were calculated by volcano plot with a fold change (FC) threshold of ≥|2| and Wilcoxon rank test *p*-value threshold of 0.1. The red circles represent the seven upregulated metabolites above the thresholds for the HCRs vs. LCRs comparison.

The unsupervised principal component analysis (PCA) displayed the overall distribution based on the 51 annotated metabolites among all samples (Supplementary Figure S1). Apart from two HCR rats, the first PC separates the HCR and LCR samples with a variance of 78.2% indicating a good analytic stability and metabolic diversity among the two groups. Clear dynamic changes for each metabolite can also be observed by hierarchical cluster analysis (Supplementary Figure S2). Seven metabolite (Table S1, Figure 2) differences with a *p*-value threshold of <0.1, fold change (FC) ≥ |2|between HCRs compared to LCRs were detected. The seven serum metabolites were upregulated in the HCR compared to LCR rats. Glycine, alanine, lysine, tetradecenoylcarnitine, dicarboxypalmitoylcarnitine, 3-hydroxy-octadecanoylcarnitine, hydroxy-octadec-2-enoylcarnitine revealed to be differentially elevated in HCR compared to LCR rats.

### 2.3. Ace2 and Body Weight Correlations

**Figure 3:**
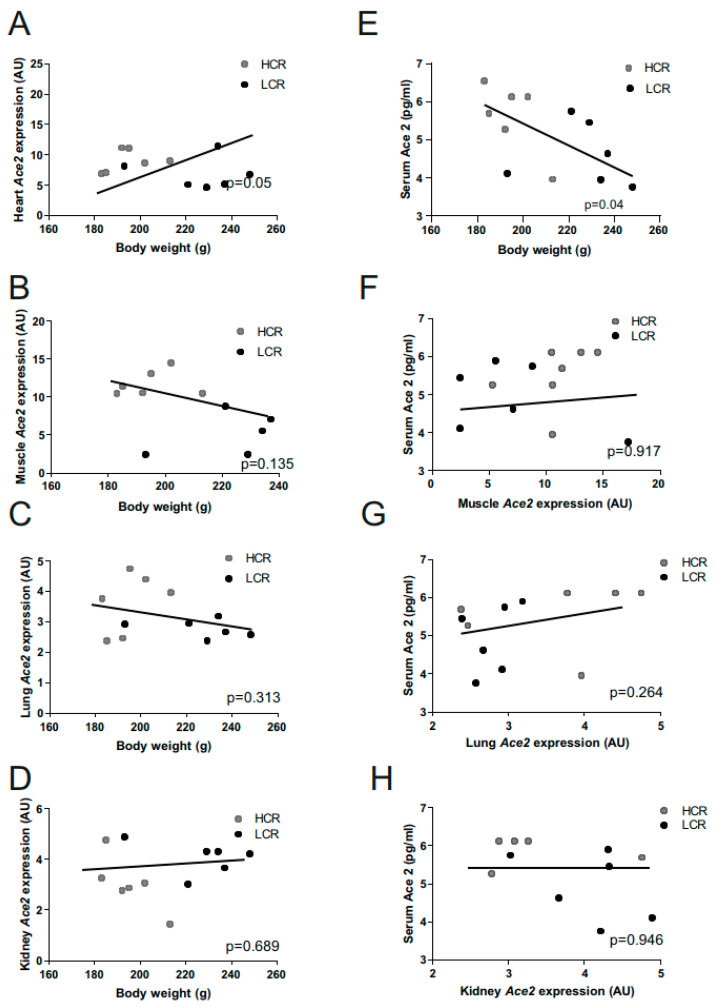
Correlation between body weight and ACE2 expression and ACE2 serum concentration. The univariate linear regression analyses reveal significant correlations between the body weight and (**A**) heart muscle ACE2 mRNA expression but no correlation with ACE2 expression (**B**) in muscle (**C**) in lungs or (**D**) kidneys were found. Serum ACE2 concentrations correlate with body weight (**E**) but not with muscle ACE2 expression (**F**), lung ACE2 expression (**G**) nor kidney ACE2 expression (**H**) in 12 untrained high- and low-capacity rats. *p*-values are given.

We analyzed the correlation of body weight with ACE2 expression in different tissues and serum in sedentary HCRs and LCRs. Univariate linear regression analyses revealed a significant positive correlation between cardiac ACE2 mRNA with body weight (Figure 3A) and a significant negative correlation of circulating ACE2 serum levels to body weight (Figure 3E). Muscle, kidney, and lung ACE2 mRNA levels were not associated with body weight (Figure 3B–D) or serum ACE2 levels (Figure 3F–H).

### 2.4. Effects of Exercise Training on Ace2 Muscle Expression

Aerobic interval training for 4 weeks led to a significant increase in exercise capacity in both HCRs and LCRs, indicating that exercise training was effective (Table 1).

**Figure 4:**
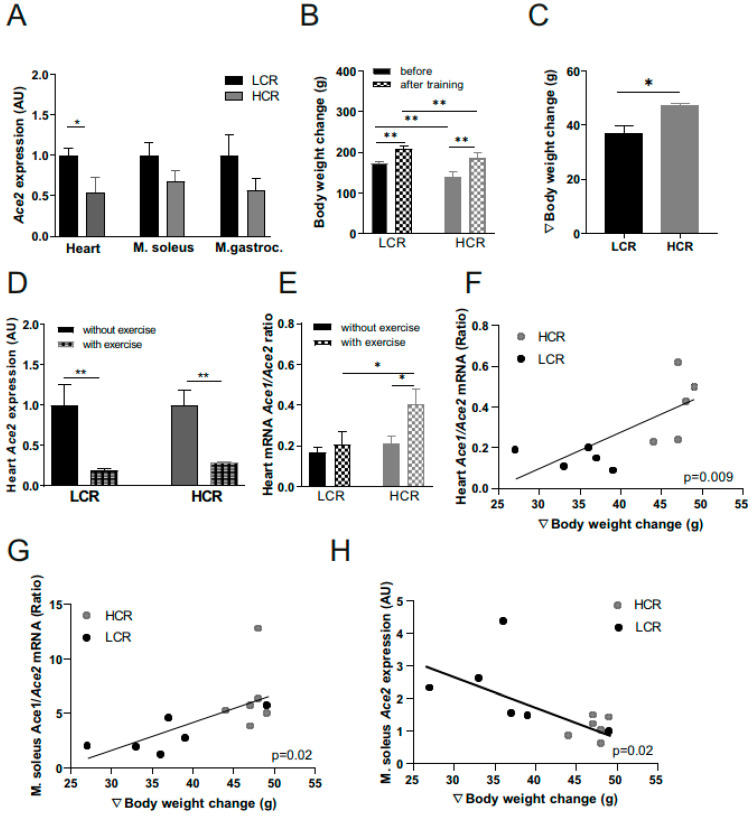
ACE2 and ACE1/ACE2 ratio mRNA expression in skeletal muscle and heart of low- versus high-capacity rats and in response to 4 weeks of interval training. The effects of a previously reported high-intensity aerobic interval training [15] was investigated in 19-week-old female high-capacity runners (HCRs, n = 5) and compared to low-capacity runners (LCRs, n = 5). (**A**) Baseline ACE2 mRNA expression differences in heart and different skeletal muscles (Musculus soleus, M. soleus; Musculus gastrocnemius, M. gastroc.) between LCR and HCR rats. (**B**) Effects of a 4 weeks of intensive aerobic interval training on: body weight summarized as body weight change; (**C**) and the heart ACE2 mRNA expression in LCRs and HCRs (**D**). Effects of 4 weeks of intensive aerobic interval training on the ACE2/ACE1 mRNA ratio in the heart (**E**) as well as the correlation of heart (**F**) and *M. soleus* (**G**) ACE1/ACE2 ratio to body weight change. Correlation analysis of ACE2 expression in M. soleus and body weight change (**H**). mRNA level was normalized to 18sRNA. Results are expressed as the means ± SE. *, *p* < 0.05; **, *p* < 0.01. Differences among the groups were performed using t-test or linear regression analysis with GraphPad Prism 9 Software (Jandel Scientific, San Rafael, CA, USA).

Furthermore, the increase in mass indicated a higher increase in the amount of muscle (Figure 4B,C). As observed for sedentary animals, the ACE2 expression in the heart and muscle was again higher in the exercise-trained LCRs compared to the exercise-trained HCRs (Figure 4A). Interestingly, in both strains, 4 weeks of exercise training significantly reduced the ACE2 mRNA expression in the heart (Figure 4D), but not in other tissues (not shown). The cardiac ACE1/ACE2 mRNA ratio was significantly elevated after aerobic interval training in HCR rats only (Figure 4E). Moreover, the body weight change during training (Figure 4C) was correlated with an ACE1/ACE2 ratio in the heart and M. soleus (Figure 4F,G). The ACE2 mRNA level is negatively associated with the body weight change (Figure 4H). These results suggest that a more pronounced response to exercise is connected to stronger effects on ACE.

## 3. Discussion

We showed in this analysis, for the first time, that a lower ACE2 expression in tissue, higher ACE levels in serum, and metabolome differences are related to high intrinsic aerobic exercise capacity. Exercise training reduces ACE2 expression in heart and muscle and the strength of this effect depends on the strength of the response to exercise. These results lend support to the suggestion of greater SARS-CoV-2 resistance in physically fit and active individuals.

In the ongoing COVID-19 pandemic, it is surprising how little is known about the role of exercise in the susceptibility for infection and whether regular physical activity modulates COVID-19 disease severity. Comorbid conditions such as hypertension, obesity, diabetes, and chronic pulmonary diseases are associated with more admissions to intensive care units and more severe disease courses [17,18]. These conditions are frequently associated with lower regular physical activity and cardiorespiratory fitness. However, it remains open whether exercise protects against SARS-CoV-2 infections or may attenuate the severity of COVID-19. It is further unclear if exercise training is beneficial [19].

Regular physical exercise promotes a stronger immune response and decreases susceptibility to pathogenic microorganisms, including viruses. In contrast, the infection burden is high among high-performance athletes [20] and may create an open window for COVID-19 infections. Whether physical exercise affects the propagation of SARS-CoV-2 or the incidence and course of COVID-19 is currently unknown. In this context, it has been suggested that a higher cardiorespiratory fitness in physically active individuals may confer some innate immune-protection against COVID-19 by attenuating the cytokine storm syndrome [21].

ACE2, the SARS-CoV-2 cell surface receptor [4], may provide a mechanistic link between exercise and the likelihood to be infected by the virus. Indeed, ACE2 is upregulated in adipocytes of people with obesity and diabetes and may explain why these diseases are frequent comorbidities of COVID-19 infections [22]. Moreover, high-intensity interval exercise—and to a lesser extent—moderate intensity continuous exercise have been shown to acutely increase plasma ACE2 concentrations in humans [10]. In contrast, in young healthy men, an intensive 4-week exercise program led to significantly reduced ACE2 serum concentrations [23]. However, the effects of exercise on ACE2 expression in tissues relevant to SARS-CoV-2 infections (e.g., lung, heart) have not been studied in humans. In mice, high-intensity interval training led to cardiac remodeling through modulations of the heart´s renin–angiotensin system including a decreased expression of the angiotensin type 2 receptor [24]. On the other hand an unhealthy high caloric carbohydrate-rich diet was associated with higher ACE2 mRNA expression in the heart [24].

Here, we show that rats genetically selected for a high or low running capacity [15] were significantly different for both circulating ACE2 and ACE2 tissue expression. We found that the HCR rats are leaner with significantly lower ACE2 mRNA expression in the lung, heart, skeletal muscle, and kidney compared to LCR rats. Indeed, the ACE2 expression in the heart correlates with body weight suggesting that with increasing body weight, the risk for cellular SARS-CoV-2 entry increases. It is noteworthy that the lung ACE2 expression was ~60% lower in HCR compared to LCR rats. Of course, our data do not allow drawing conclusions for an increased COVID-19 infection risk, but may provide a hypothesis for a mechanism underlying increased COVID-19 susceptibility and more fatal outcomes in patients with lower exercise capacity, often associated with obesity.

We also find seven metabolites which were different between LCRs and HCRs. The volcano plot combines results from the fold change analysis and Wilcoxon rank test into one single graph which allows users to intuitively select significant features based on either biological significance, statistical significance, or both. Alanine, glycine, lysine, tetradecenoylcarnitine, dicarboxypalmitoylcarnitine, 3-hydroxy-octadecanoylcarnitine, and hydroxy-octadec-2-enoylcarnitine were revealed to be differentially circulating. Interestingly, amino acid neurotransmitters, including glutamate, phenylalanine, tyrosine, alanine, and glycine, underlie the majority of the excitatory and inhibitory neurotransmissions in the nervous system, and acute exercise has been shown to modulate their concentrations [25]. Alanine and glycine are upregulated in HCR rats and may indicate an excitatory neurotransmission in the nervous system. Recent metabolomic and genetic studies confirmed a strong association of type 2 diabetes (T2D) with decreased glycine levels in human subjects [26,27,28]. Furthermore, it has been shown that glycine has beneficial effects against the proinflammatory cytokine secretion induced by SARS-CoV-2 infection [13,14]. Interestingly, the plasma levels of glycine appear to be significantly decreased in ACE2 null mice [12], suggesting that the activation of ACE2 would increase the plasma glycine levels. It was proposed that glycine itself should be used in the treatment of COVID-19 patients due to its anti-inflammatory, anti-oxidative, neurological functions, and role in metabolic regulation [13,14]. Ivermectin, as a partial agonist of glycine-gated chloride channels, was also recently suggested to interfere with the COVID-19 cytokine storm by inducing the activation of glycine receptors [13,14].

In addition, HCR rats have a higher circulating ACE2 compared to LCR rats, which has been considered a COVID-19 protective factor [6]. It remains unclear why HCR rats have higher circulating ACE2 despite having a lower ACE2 tissue expression compared to LCR rats. We cannot exclude that tissues other than heart, lung, muscle, and kidneys contribute to these higher circulating levels. The negative correlation of ACE2 serum concentrations and body weight observed in our study supports the suggested role of adipose tissue as a site of ACE2 modulation [22]. In addition, the post-translational modifications and shedding of the ACE2 cell surface receptor differences between the LCR and HCR groups may contribute to the observed differences. It has been demonstrated that high amounts of circulatory ACE2 block the SARS-CoV protein binding to its receptor [29]. This is in agreement with some studies announcing that higher ACE2 levels may protect from COVID-19 onset [30,31]. The higher primary level of ACE2 in Asian females than in men in line with a higher case of fatality in men and lower severity in females suggest a protective effect of ACE2 on COVID-19 [31]. We further sought to determine the effects of an intensive aerobic exercise training program over four weeks on five consecutive days per week on ACE2 tissue expression. Independently of the rat strain, the ACE2 expression was reduced by approximately 80% in the heart. However, even after the exercise intervention, HCR rats had a significantly lower ACE2 mRNA expression in the heart than LCR rats and a trend to lower skeletal muscle ACE2 expression. Interestingly, in the heart, the ACE1/ACE2 mRNA ratio was significant elevated after training and associated with body weight change. A high ratio could explain the increased protection against endothelial dysfunctions and vascular disorders, probably leading to decreased capillary permeability, coagulation, fibrosis, and apoptosis in the alveolar cells, and decreasing the lung damage sparked by tSARS-CoV-2 [32]. We suggest that aerobic interval training lowers ACE2 activity, which may lead to a higher ratio.

## 4. Materials and Methods

### 4.1. Animals

All animal procedures were approved by the Animal Welfare Committee of the University of Leipzig and Jena, Germany. Animals were handled and housed in accordance with the National Institutes of Health (NIH) guidelines. The generation of the high-/low-capacity rat (HCRs/LCRs) model of high and low intrinsic aerobic capacity was described previously [16]. Briefly, bidirectionally selected lines were generated from a founder population of 80 male and 88 female N:NIH stock rats based on intrinsic aerobic treadmill running capacity. Thirteen families for each line were set up for a within-family rotational breeding paradigm that keeps the inbreeding at <1% per generation. At each generation, young adult rats (11 weeks of age) were tested for their inherent ability to perform forced speed-ramped treadmill running until exhaustion. This test was performed daily over five consecutive days. The greatest distance in meters achieved out of the five trials was considered the best estimate of an individual’s aerobic exercise capacity [16]. The highest scoring female and male from each of the 13 families were selected as breeders for the next generation of high-capacity runners (HCRs). The same process was used with lowest scoring females and males to generate low-capacity runners (LCRs). The female HCR and LCR rats (generations 33 and 36, 17–20 weeks of age) used for this investigation were housed in pairs in a temperature-controlled environment with a 12 h light/dark cycle.

At the age of 19 weeks, 12 sedentary female (6 LCR and 6 HCR) rats were sacrificed and heart, muscle, kidney, subcutaneous (SC) and epigonadal (EPI) adipose tissue, and the lung were quickly removed and fixed in liquid nitrogen. Body weights were measured and serum was collected.

### 4.2. Exercise Training Intervention

In a separate cohort of 10 age-matched female rats, we assessed exercise capacity. Animals were accommodated to the treadmill at an age of 12–13 weeks by allowing them to be on the treadmill for 15 min at low speed at three single days. Thereafter, the animals were tested for their exercise capacity using a ramped protocol at a 25° incline. After 15 min of warm-up at 13 m/min (HCRs) or 4 m/min (LCRs), the treadmill speed was increased by 1.8 m/min every two minutes until the animals were exhausted (resting at the stimulation unit for three (HCRs) or six (LCRs) times for five seconds or the equivalent at one exercise level). This was repeated another two times after one day of rest. The mean (HCRs) or the maximum (LCRs) of the three attempts was considered as the maximal exercise capacity and the last four steps were subtracted to account for anaerobic exercise capacity. The determined aerobic exercise capacity (100%) was used to calculate the speed for the training sessions. Starting at the age of 14 weeks, the animals exercised for four weeks on five consecutive days per week using a protocol with a 15 min warm up (45% of aerobic exercise capacity) and 90 min interval training at 85% (8 min) and 55% (2 min) of aerobic capacity. If animals fully completed the exercise sessions of at least 3 out of the 5 days during one week, 1.2 m/min were added to their maximal aerobic exercise capacity and the protocol was recalculated. One day after the last training session organs were harvested.

### 4.3. RNA Isolation, Ace 1 and Ace 2 Gene Expression, and Ace2 Serum Concentrations

RNA isolation and quantitative real-time PCR was performed as previously described [33]. Specific mRNA expression was calculated relative to *18sRNA* which was used as an internal control due to its resistance to glucose-dependent regulation [34].

The TaqMan probe-based quantitative real-time PCR was performed using the QuantStudio 6 Flex Real-Time PCR System (Life technologies, Darmstadt, Germany). The expression of ACE1 and ACE2 was calculated by standard curve method and normalized to the expression of 18sRNA as a housekeeping gene [34]. The probes, ACE1 (Rn00561094_m1), ACE2 (Rn01416289_m1) and 18sRNA (Hs99999901_s1) were purchased from (Life technologies, Darmstadt, Germany) and span exon–exon boundaries to improve the specificity of the qPCR. ACE2 serum concentrations were measured by ELISA using rat standards according to the manufacturer’s guidelines (ACE2 ELISA; Sigma, St. Louis, MO, USA, #RAB0010-1KT). RNA from adipose tissue (AT) samples was used for Affymetrix Clariom microarray RNA analysis. The analysis of RNA integrity and RNA concentration as well as probe synthesis, hybridization and scanning was performed as previously described [35].

### 4.4. Metabolome Analysis, Data Processing, and Normalization

Mass spectrometric analysis of amino acids (ASs) and acylcarnitines (AC) was performed using a SCIEX Triple Quad 4500 System (AB SCIEX, Darmstadt, Germany) with Turbo Ion Spray Source (TIS) in combination with a HTC Pal autosampler and a Shimadzu UFLC system for flow injection analysis (FIA) according to a validated protocol [36]. Briefly, 10 µL serum was diluted 1:10 with methanol. After centrifugation, 10 µL of the supernatant was diluted with 100 µL of methanol-containing isotope-labeled standards (Chromsystems, Munich, Germany). Samples were evaporated at 70 °C for 40 min and derivatized using 60 µL of 3n butanolic-HCL (Chromsystems, Munich, Germany) at 65 °C. After evaporation, the samples were reconstituted with 150 µL of the mobile phase (1/1 *v*/*v* methanol/water) analyzed with a SCIEX 4500 quadrupole tandem mass spectrometer in multiple reaction monitoring (MRM). Concentrations of 26 AAs, 34 ACs, and free carnitine were quantified using ChemoView™ 1.4.2 software (AB SCIEX, Darmstadt, Germany). In total, 51 metabolites were detected.

The data were preprocessed and analyzed with MetaboAnalyst (v5.0, Quebec, QC, Canada [37]). To adjust for differences among samples, raw data were row-wise normalized by the median of the samples. Data were log-transformed and centered by the mean (see Supplementary Figure S1) to increase the comparability of the features. FCs were calculated as the ratios of the absolute values of changes between two group means (HCRs compared to LCRs). For measurements less than the limit of detection (LOD), LOD values were taken.

### 4.5. Data analyses and Statistics

Data are expressed as the means ± standard deviation (SD) unless stated otherwise. The unpaired Student´s t-test (normally distributed data) was performed to compare the two groups. Correlation analysis was performed by linear regression analysis. Statistical analysis was performed using the Statistical Package for Social Science, Version 20.0, (SPSS, Inc.; Chicago, IL, USA) and Prism Version 6 (GraphPad Software, Inc., La Jolla, CA, USA). *p* values < 0.05 were considered significant.

## 5. Conclusions

Taken together, we report here that physical exercise reduces the tissue expression of ACE2 and elevates the cardiac ACE1/ACE2 ratio of low and high-capacity running rats which may translate into exercise-related lower SARS-CoV-2 pathogenicity. In addition, our rat model suggests that higher intrinsic (=genetic) exercise capacity (or physical fitness) is associated with a lower ACE2 expression in COVID-19-relevant tissues, such as the lung and heart. Moreover, the metabolome profile is affected by higher intrinsic (=genetic) exercise capacity. Increased circulating glycine and ACE2 further distinguish HCR from LCR rats and may be considered in the discussion of current clinical trials aiming to increase ACE2 [6] and glycine [13] or in recently reported recombinant proteins with high affinity to the SARS-CoV-2 receptor-binding domain [38].

Our findings may be useful in future clinical studies on the effects of physical exercise or fitness level as modifiable risk factors against SARS-CoV-2 infections.

## Figures and Tables

**Table 1:** Characteristics of LCR und HCR rats after 4 weeks of exercise training.

Parameter	HCRs Trained (N = 5)	LCRs Trained (N = 5)
Body weight before training (g)	140 ± 5	172 ± 5 ***
Body weight after training (g)	202 ± 4	222 ± 7 ***
Heart weight (mg)	728 ± 32	662 ± 14 ***
Ventricle weight (mg)	691 ± 27	626 ± 14 **
Lung weight (mg)	967 ± 49	1004 ± 43
Liver weight (g)	9.85 ± 0.86	7.78 ± 0.27 ***
M. gastrocnemius weight (g)	2.52 ± 0.09	2.82 ± 0.13 **
M. soleus weight (mg)	186 ± 16	177 ± 1 **
Tibia length after training (mm)	34.7 ± 0.2	35.0 ± 0.3
**Running capacity before training**		
Mean speed (m/min)	52.0 ± 1.1	19.2 ± 0.8 ***
Maximal speed (m/min)	56.8 ± 1.2	21.7 ± 0.7 ***
**Running capacity after training**		
Mean speed (m/min)	57.9 ± 0.9	29.9 ± 0.7 ***
Maximal speed (m/min)	59.2 ± 0.8	31.9 ± 0.9 ***

HCRs, high-capacity runners; LCRs, low-capacity runners. Data are given as the mean ± SD. Differences between the HCR and LCR group are indicated as follows: **; *p* < 0.01; ***, *p* < 0.001

## Data Availability

The data presented in this study are available in article and supplementary material.

## Supplementary Materials

The following are available online at https://www.mdpi.com/article/10.3390/metabo12060548/s1, Figure S1: PCA of the LC-MS data, Figure S2: Hierarchical cluster analysis of of the LC-MS data, Figure S3: Effects of the LC-MS data before and after normalization, Table S1: Upregulated metabolites in HCR compared to LCR.
