# Supplementary material for: Intrinsic Exercise Capacity Affects Glycine and Angiotensin-Converting Enzyme 2 (ACE2) Levels in Sedentary and Exercise Trained Rats
Source: Metabolites. 2022 Jun 15;12(6):548. doi: 10.3390/metabo12060548 (PMC9228358; doi:10.3390/metabo12060548)
Supplement: Supplementary file 1 [file metabolites-12-00548-s001.zip › metabolites-1740805-supplementary.pdf]

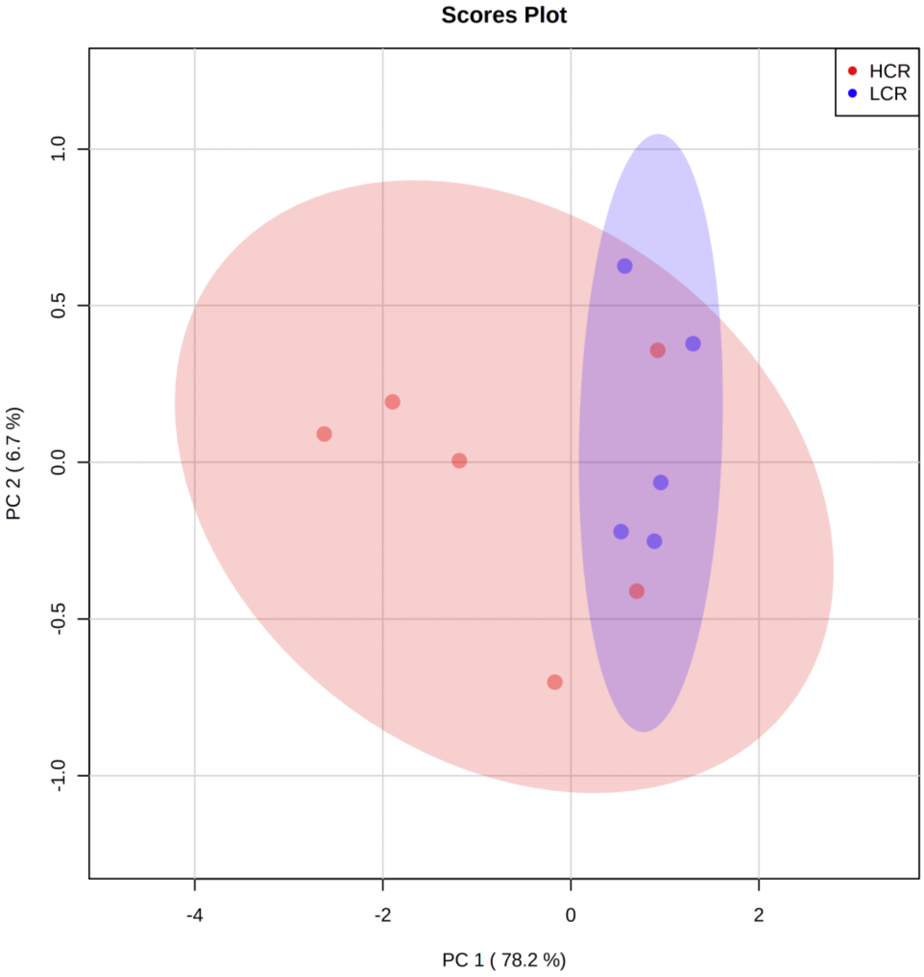

Figure S1. PCA of the LC-MS data. Unsupervised principal component analysis (PCA) of rats selectively bred for HCR (red) and LCR (blue). Except for two HCR rats, the first PCA separates the groups with a varaince of 78.2%.

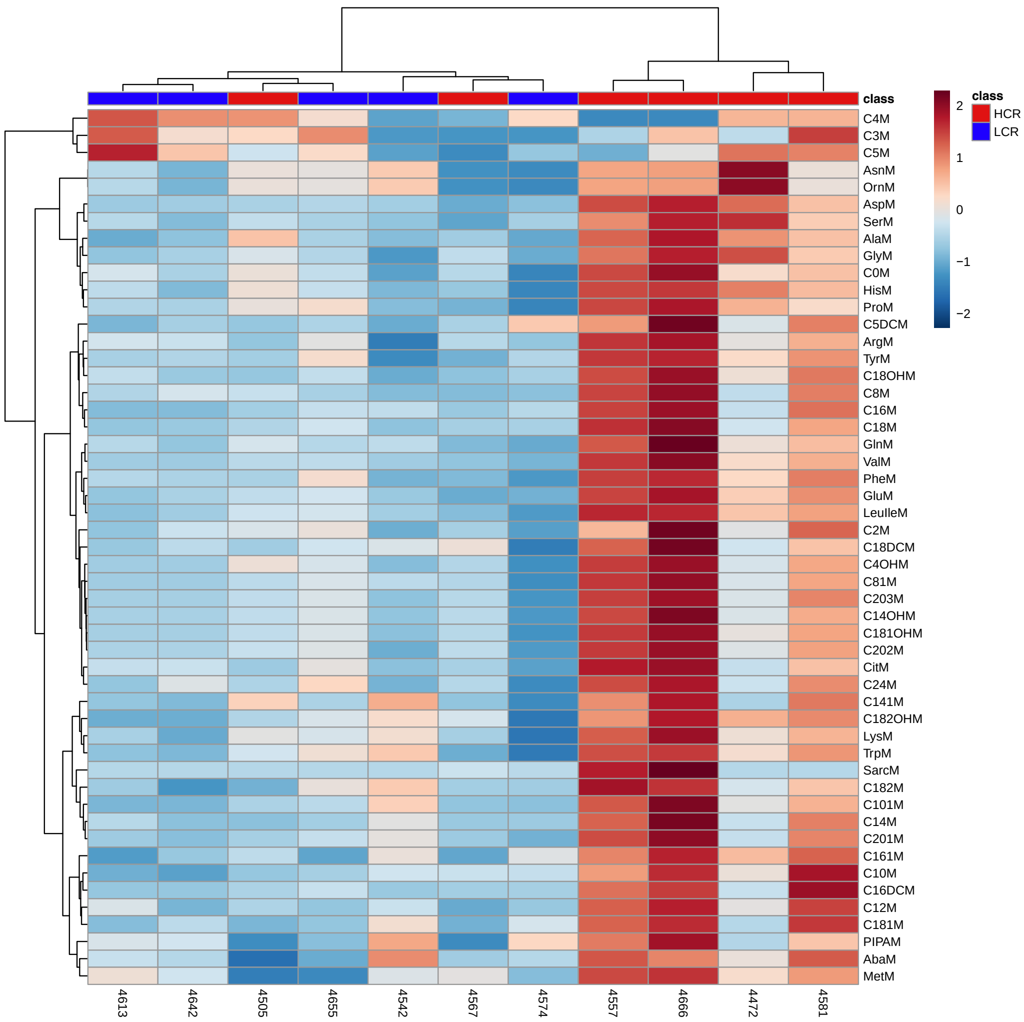

Figure S2. Hierarchical cluster analysis of of the LC-MS data. Heatmap show the differences between the metabolites for each sample and groups (red: HCR, blue: LCR). Each sample is represented in a single column and each metabolie is displayed by a single row. Red colors show higher metabolite conzentrations, while blue colors indicate reduced metabolite conzentrations. The two HCR samples 4505 and 4567 display the same behaviour as the LCR samples. Sample 4472 differs from the other HCR samples in the majority of genes. Distance measurements were performed using the Euclidean method and clustering was conducted using Ward's method with minimal variance.

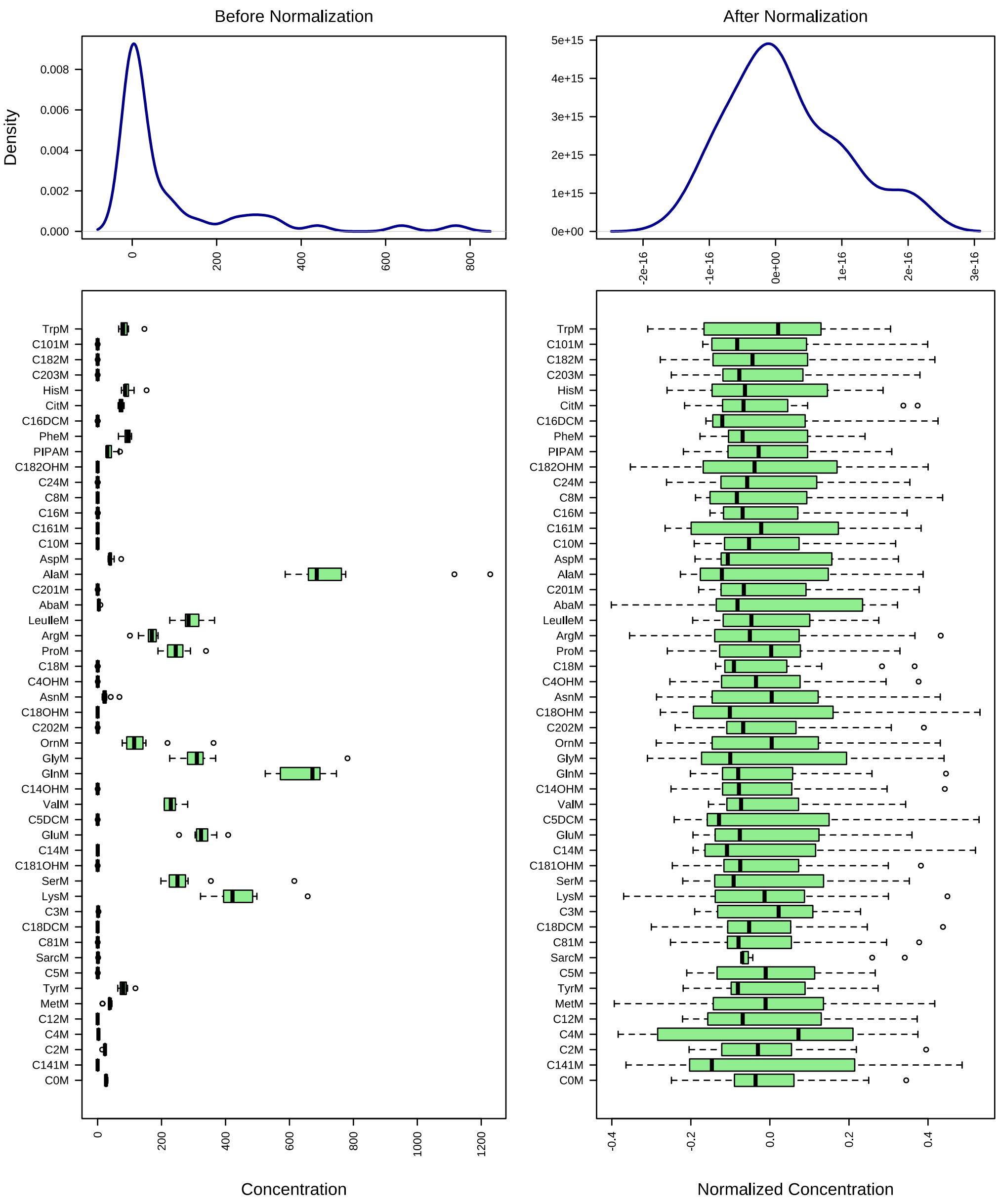

Figure S3. Effects of the LC-MS data before and after normalization. Kernel density (top) of the metabolite concentrations and box plots (bottom) before and after normalization by the sample median, log transformation and centering by the mean. The density plots are based on all samples and the boxplots show at most 50 features.

Table S1. Upregulated metabolites in HCR compared to LCR. Seven metabolites are upregulated in HCR vs. LCR considering a p-value < 0.1 and a fold change (FC) ≥ |2|.

Metabolites	FC	log2(FC)	p-value	-log10(p-value)
AlaM	2.3084	1.2069	0.0026608	2.575
GlyM	2.6304	1.3953	0.005358	2.271
C182OHM	2.0067	1.0048	0.022074	1.6561
LysM	2.0031	1.0022	0.042331	1.3733
C16DCM	2.0425	1.0304	0.056574	1.2474
C18OHM	2.4439	1.2892	0.065056	1.1867
C141M	2.0134	1.0096	0.087503	1.058

AlaM, Alanine; GlyM, Glycine; LysM, Lysine; C141M, Tetradecenoylcarnitine; C16DCM, Dicarboxypalmitoylcarnitine; C18OHM, 3-Hydroxy-octadecanoylcarnitine; C182OHM , Hydroxy-octadec-2-enoylcarnitine.
